# Comprehensive identification of the full-length transcripts and alternative splicing related to the secondary metabolism pathways in the tea plant (*Camellia sinensis*)

**DOI:** 10.1038/s41598-019-39286-z

**Published:** 2019-02-25

**Authors:** Dahe Qiao, Chun Yang, Juan Chen, Yan Guo, Yan Li, Suzhen Niu, Kemei Cao, Zhengwu Chen

**Affiliations:** 1 Tea Research Institute, Guizhou Academy of Agricultural Science, Guiyang, 550006 Guizhou China; 2 College of tea science, Guizhou University, Guiyang, 550025 Guizhou China

## Abstract

Flavonoids, theanine and caffeine are the main secondary metabolites of the tea plant (*Camellia sinensis*), which account for the tea’s unique flavor quality and health benefits. The biosynthesis pathways of these metabolites have been extensively studied at the transcriptional level, but the regulatory mechanisms are still unclear. In this study, to explore the transcriptome diversity and complexity of tea plant, PacBio Iso-Seq and RNA-seq analysis were combined to obtain full-length transcripts and to profile the changes in gene expression during the leaf development. A total of 1,388,066 reads of insert (ROI) were generated with an average length of 1,762 bp, and more than 54% (755,716) of the ROIs were full-length non-chimeric (FLNC) reads. The Benchmarking Universal Single-Copy Orthologue (BUSCO) completeness was 92.7%. A total of 93,883 non-redundant transcripts were obtained, and 87,395 (93.1%) were new alternatively spliced isoforms. Meanwhile, 7,650 differential expression transcripts (DETs) were identified. A total of 28,980 alternative splicing (AS) events were predicted, including 1,297 differential AS (DAS) events. The transcript isoforms of the key genes involved in the flavonoid, theanine and caffeine biosynthesis pathways were characterized. Additionally, 5,777 fusion transcripts and 9,052 long non-coding RNAs (lncRNAs) were also predicted. Our results revealed that AS potentially plays a crucial role in the regulation of the secondary metabolism of the tea plant. These findings enhanced our understanding of the complexity of the secondary metabolic regulation of tea plants and provided a basis for the subsequent exploration of the regulatory mechanisms of flavonoid, theanine and caffeine biosynthesis in tea plants.

## Introduction

The tea plant [*Camellia sinensis* (L.) O. Kuntze] is an important commercial crop that is widely grown throughout the world^1^. The leaves of tea plants are the main raw material for tea production. Tea is one of the most popular non-alcoholic beverages, and its rich flavors and health benefits primarily stem from the secondary metabolite components^2–4^. Flavonoids, theanine and caffeine are the three major secondary metabolites in tea plants^5^. The flavonoids primarily give tea an astringent taste, theanine contributes to the umami taste, and caffeine offers a bitter taste^6^. An in-depth exploration of the metabolic mechanisms of these secondary metabolites will help us to improve the quality of tea.

Flavonoids are phenylalanine-derived, and include flavanones, flavones, flavonols, isoflavones, flavin-3-ols and anthocyanidins^7^. The flavin-3-ols, which are also known as catechins in tea plants, consist of a mixture of (+)-catechins (C), (+)-gallocatechin (GC), (-)-epicatechin (EC), (-)-epigallocatechin(EGC), (-)-epicatechin-3-gallate (ECG), and (-)-epigallocatechin-3-gallate (EGCG), which can be divided into non-gallated catechins (C, GC, EC and EGC) and gallated catechins (ECG and EGCG)^8^. The non-gallated catechins are synthesized through the phenylpropanoid and flavonoid pathways, and previous studies have shown that thirteen genes are involved in these pathways, including phenylalanine ammonia-lyase (*PAL*), cinnamate-4-hydroxylase (*C4H*), 4-coumarate-CoA ligase (*4CL*), chalcone synthase (*CHS*), chalcone isomerase (*CHI*), flavanone 3-hydroxylase (*F3H*), flavonol synthase (*FLS*), flavonoid 3′-hydroxylase (*F3′H*), flavonoid 3′,5′-hydroxylase (*F3′5′H*), dihydroflavonol-4-reductase(*DFR*), anthocyanidin synthase (*ANS*), leucoanthocyanidinreductase (*LAR*) and anthocyanidin reductase (*ANR*)^9^. The galloylated catechins are synthesized under the galloyl-1-O-β-D-glucosyltransferase (UGGT) and epicatechin:1-O-galloyl-β-D-glucose O-galloyltransferase (ECGT)^5^. Although most of the genes involved in these pathways have been identified, many of them are in the form of multiple gene families^10–13^, and thus, the basic regulatory mechanisms of the catechins biosynthesis in tea plants are still unclear. Theanine is a special non-coding amino acid in tea plants, which can account for more than half of the total free amino acids in the plant^14^. Theanine is synthesized from glutamic acid and ethylamine by theanine synthetase (TS). This amino acid is first synthesized at the roots and then transported to various parts of the plant^5,15^. Additionally, alanine transaminase (ALT), arginine decarboxylase (ADC), glutamine synthetase (GS), glutamate synthase (GOGAT) and glutamate dehydrogenase (GDH) are also involved in the process^6^. Although this pathway has been studied extensively, the *TS* gene was not identified until recently^5^. Caffeine (1, 3, 7-trimethylxanthine) is a purine alkaloid, that has been widely used as a stimulant and an ingredient indrugs^6^. In tea plants, caffeine primarily exists in young leaves, and with the development of leaves, the contents of caffeine markedly decreases^6,16^. Caffeine is synthesized from xanthosine via the purine biosynthesis pathway and purine modification steps. In this process, approximately eight key genes are involved, including adenosine nucleosidase (Anase), adenine phosphoribosyltransferase (APRT), AMP deaminase (AMPD), IMP dehydrogenase (IMPDH), 5′-nucleotidase (5′-Nase), xanthosine methyltransferase (7-NMT), theobromine synthase (7-methylxanthine methyltransferase, MXMT) and caffeine synthase (3, 7-dimethylxanthine methyltransferase, TCS)^16,17^. Moreover, based on the analysis of genomic sequencing data, it was determined that the caffeine synthetic pathway in tea plants evolved independently from that in coffee^5,18^. Similar to the catechins synthetic pathway, the genes involved in theanine and caffeine synthesis also present a multigene family^5,19^.

Alternative splicing (AS) is a crucial post-transcriptional regulatory mechanism that exists widely in eukaryotes^20,21^. Through AS, the same gene can generate multiple mRNAs, thus, AS can significantly increase the diversity and complexity of the transcriptome and proteome^21,22^. AS events can be divided into the following five categories: intron retention (IR), exon skipping (ES), alternative 3′ splice site (A3SS), alternative 5′ splice site (A5SS) and mutually exclusive exon (MXE)^23^. IR is the most common form of AS in plants, accounting for 32.2% of the AS events in polyploid cotton^24^, 37.55% in cucumber^25^ and approximately 40% in Arabidopsis^26^ and maize^27^. Previous studies have shown that the alternatively spliced isoforms have tissue or temporal-based preferential expression characteristics, and they can also be influenced by environmental conditions^24,25,28^. Differential AS events have been demonstrated to play vital roles in plant development, growth and stresses responses^29,30^. In tea plants, the types and contents of the secondary metabolites also vary depending on the tissue type and in different periods as well as in different environments^6,31,32^, which is similar to the expression patterns of alternatively spliced isoforms. This phenomenon suggests that the AS events of related genes may regulate the secondary metabolism of tea plant and may thus affect the tea quality. This hypothesis has been demonstrated in tea plants. For example, the two splicing forms of the terpene synthase gene *CsLIS/NES-1* and *CsLIS/NES-2*, not only exhibit distinctly different expression patterns and subcellular localization, but are also involved in linalool and nerolidol biosynthesis in tea plants, respectively^33^. Additionally, a recent study using full-length transcriptome sequencing revealed AS events in the *CHS*, *LAR*, *GS*, *5′-Nase* and *TCS* genes^7^. In another study, certain IR events of the *CHI* and *DFR* genes were identified based on the analysis of genomic and transcriptome sequencing data, which also showed that the AS events were correlated to tissue types and genotypes in tea plants^11^. Therefore, the identification of AS events that are related to the secondary metabolic pathways are of great significance for an in-depth understanding of the secondary metabolic regulation in tea plants.

Recently, the genomic data of two primary types of cultivated tea plant varieties have been published^5,18^, which provide valuable information for investigations of the gene structure and transcriptome of the tea plants. In this study, single molecular real-time (SMRT) sequencing and RNA-seq analysis were performed to identify the key genes involved in flavonoid, theanine and caffeine biosynthesis. Based on the obtained full-length transcripts and the reference genome sequences, the AS events of these key genes were identified along with the expression patterns of the AS events in various tissues. Our results provide a foundation for future studies and insight into the role of AS in flavonoid, theanine and caffeine metabolism in tea plants.

## Results and Discussion

### General properties of SMRT sequencing and DET identification

To further explore the regulatory mechanism of secondary metabolism of the tea plant, equal amounts of total RNA from four tissues (apical bud, first leaf, second leaf, and roots) were pooled together to obtain full-length transcripts for SMRT sequencing. Additionally, three biological replicates of apical bud (Bud-1, -2, -3) and second leaf (SL-1, -2, -3) tissues were used for the RNA-seq analysis (Supplementary Fig. S1). Based on the PacBio Sequel platform, three SMRT cells were sequenced with the 1–6 k cDNA size. A total of 1,388,066 reads of insert (ROIs) were generated with an average length of 1,762 bp, and the amount of ROIs obtained in this study was much higher than that obtained in a previous study based on the PacBio RSII platform (Supplementary Table S1)^7^. Among of the ROIs, more than 54% (755,716) of them were full-length non-chimeric (FLNC) reads (Table 1). The RS_IsoSeq module of the SMRT analysis (v2.3.0) was used to cluster the FLNC reads, and a total of 427,219 consensus isoforms were obtained, of which 48,704 were high-quality consensus transcript sequences. The ICE clustering results are shown in Table 2. The low-quality isoforms then were corrected using the RNA-seq data. Ultimately, a total of 93,883 non-redundant transcripts were obtained (each transcripts was named as PB.xxxxx.x) after mapping to the reference genome (http://pcsb.ahau.edu.cn:8080/CSS/). Based on BUSCO^34^, 92.7% (S:38.6%, D:54.1%) of the 1,440 expected embryophytic genes were identified as complete, and 3.2% and 4.1% were identified as fragmented and missing, respectively, which indicated that the integrity of the transcriptome obtained in this study was high (Supplementary Fig. S2).

**Table 1:** The statistical analysis of the PacBio single-molecule long-read sequencing.

Samples	cDNA Size	Reads of Insert	Number of five prime reads	Number of three prime reads	Number of poly-A reads	Number of filtered short reads	Number of non-full-length reads	Number of full-length reads	Number of full-length non-chimeric reads	Average full-length non-chimeric read length	Full-Length Percentage (FL%)
Tea	1–6 K	1,388,066	906,872	940,779	924,858	85,610	542,411	760,045	755,716	1,763	54.76%

**Table 2:** The results of Iterative Clustering for Error Correction (ICE) clustering analysis.

Samples	Size	Number of consensus isoforms	Average consensus isoforms read length	Number of polished high-quality isoforms	Number of polished low-quality isoforms	Percent of polished high-quality isoforms(%)
Tea	0 to 1 kb	135,930	669	10,652	124,960	7.84%
Tea	1 to 2 kb	127,406	1,478	17,909	109,422	14.06%
Tea	2 to 3 kb	105,069	2,459	14,729	90,330	14.02%
Tea	3 to 6 kb	57,590	3,702	5,384	52,204	9.35%
Tea	Above 6 kb	1,224	6,450	30	1,194	2.45%
Tea	All	427,219	1,775	48,704	378,110	11.40%

**Figure 1:**
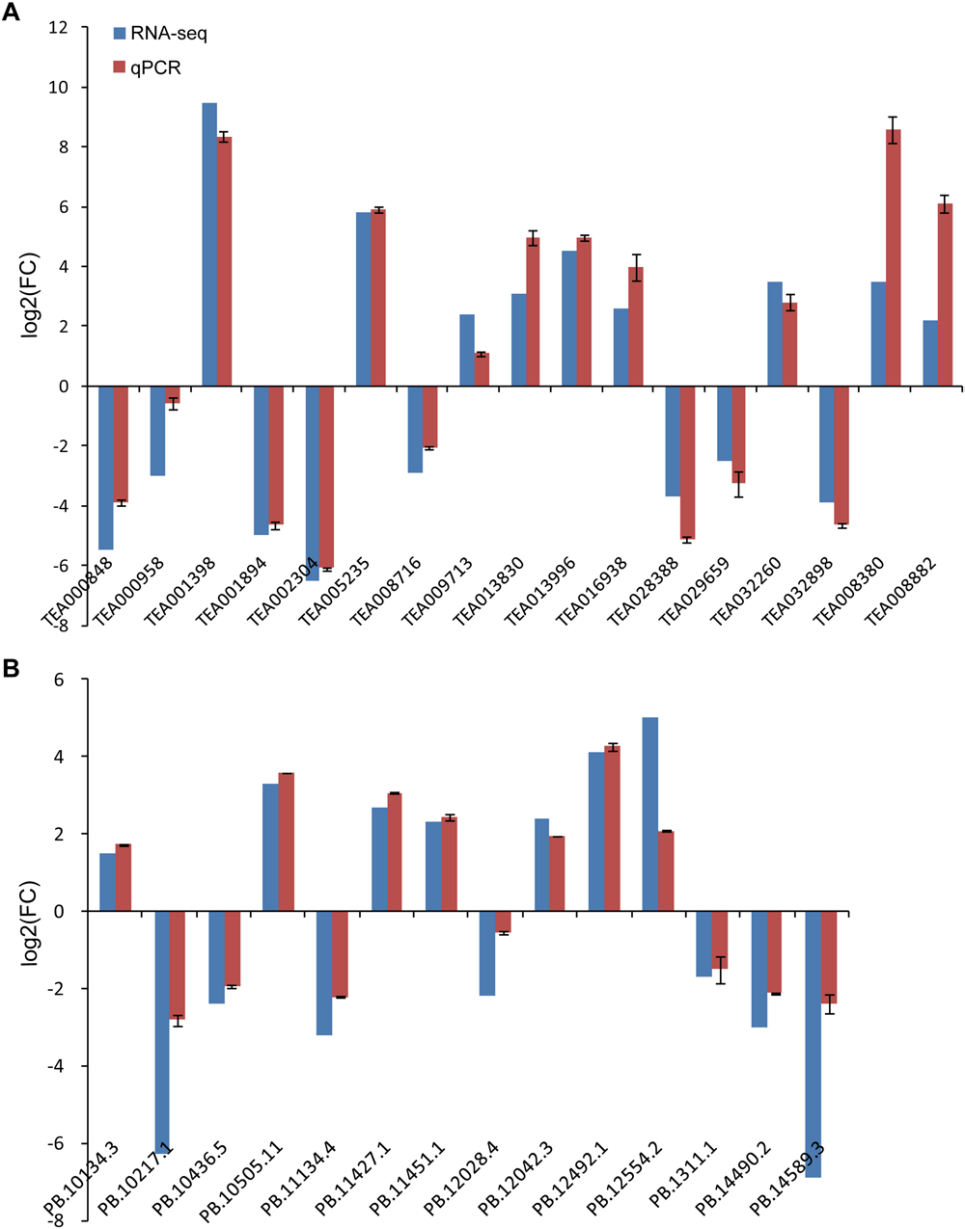
qPCR validation of the differentially expressed genes (**A**) and the differentially expressed transcripts (**B**).

The expression levels of the transcripts were calculated based upon the fragments per kilobase of transcript per million fragments mapped (FPKM) values. The transcripts with differential expressions between Bud and SL tissues were also identified. In total, 7,650 differentially expressed transcripts (DETs) from 5,354 differentially expressed genes (DEGs) were identified, which accounted for 8.15% of all transcripts identified in this study. Meanwhile, among the DETs, 3,885 and 3,765 transcripts were up-regulated and down-regulated in SL, respectively (Supplementary Fig. S3, Supplementary Table S2). Seventeen DEGs and fourteen DETs were selected for qPCR analysis, and the expression profiles of them were similar to the RNA-seq results, which demonstrated that the RNA-Seq data were reliable (Fig. 1).

### Alternative splicing identification

**Figure 2:**
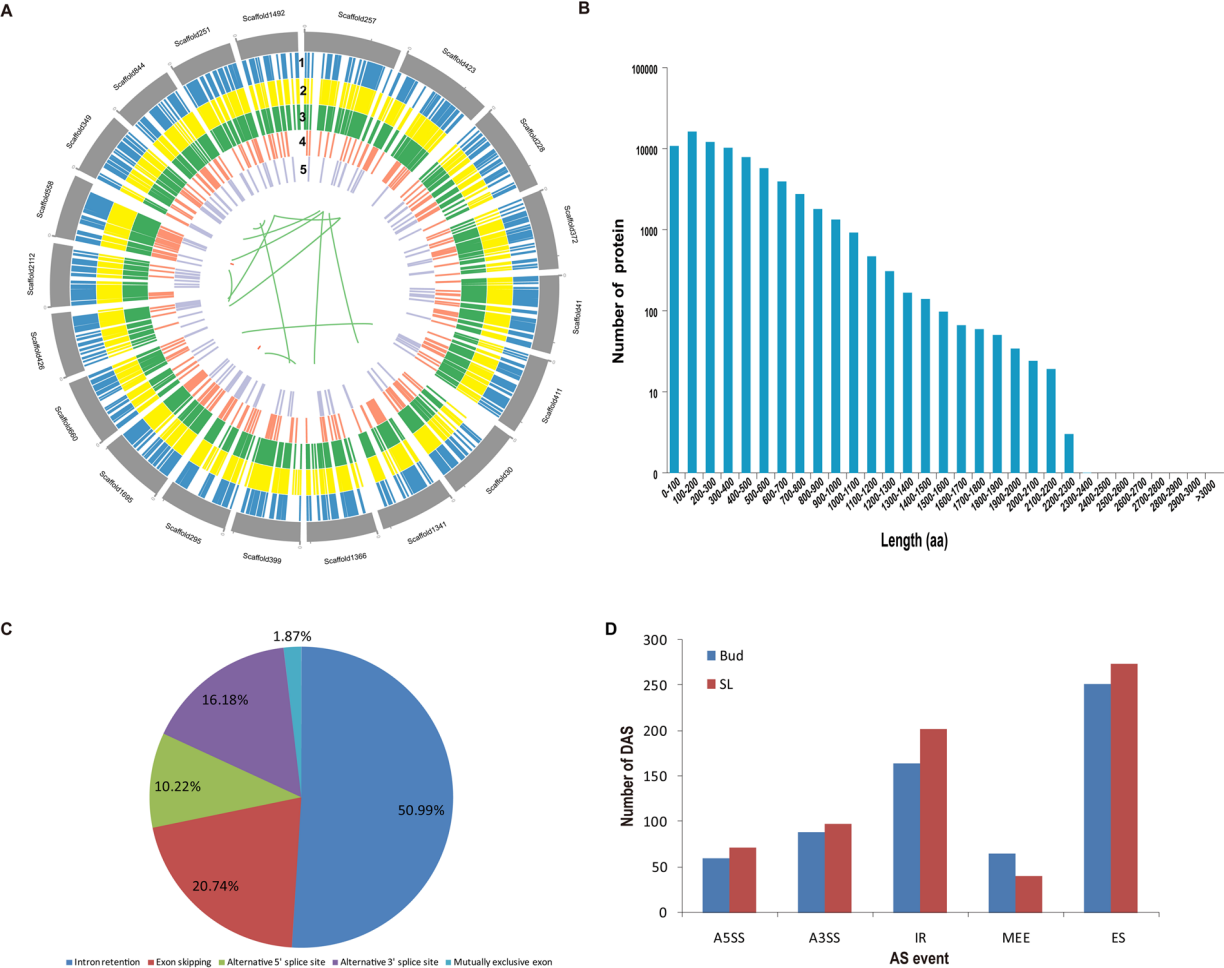
Characteristics of the transcripts and alternative splicing events in the tea plant. (**A**) Circos visualization of the transcriptomic profiles. The largest twenty scaffolds are shown at the outer circles. The 1 to 5 display simple sequence repeats (SSRs), all transcripts, new identified transcripts, transcriptional factors (TFs) and fusion transcripts, respectively. (**B**) The coding protein length distribution of the predicted CDS. (**C**) The summary of alternative splicing events. (**D**) The differential alternative splicing (DAS) events in Bud and SL.

The 93,883 non-redundant transcripts were compared against the gene set of tea plant reference genome (http://pcsb.ahau.edu.cn:8080/CSS/), and 30,866 gene loci were detected, including 11,602 predicted novel genetic loci. Among the non-redundant transcripts, 87,395 were new isoforms. The transcripts with a sequence length greater than 500 bp were screened, and an SSR analysis was performed using MISA software. A total of 90,324 sequences (195,858,944 bp) were examined, and 127,845 SSRs were identified from 60,509 sequences. Because the reference genome of the tea plant has not yet revealed the chromosome level, the largest twenty scaffolds identified in this study were selected to preliminarily show the distribution of the SSRs and transcripts in the tea plant genome (Fig. 2A). Additionally, the coding sequences and their corresponding amino acid (aa) sequences of the new transcripts were also predicted using the TransDecoder (v3.0.0). A total of 74,795 open reading frames (ORFs) were identified, which included 55,506 complete ORFs. The distribution of the corresponding amino acid sequence lengths of the ORFs is shown in Fig. 2B.

AStalavista^35^ was used to identify the alternative splicing (AS) events from the transcripts and a total of 28,980 AS events were identified (Supplementary Table S3). Among the five main types of alternative splicing, intron retention (IR) predominated, accounting for 50.99% of the alternatively spliced transcripts, followed by exon skipping (ES, 20.74%), alternative 3′ splice site (A3SS, 16.18%), alternative 5′ splice site (A5SS, 10.22%) and mutually exclusive exon (MXE, 1.87%) (Fig. 2C, Supplementary Table S3). This finding is consistent with previous studies in various plants, such as *Arabidopsis*^26^, maize^27^, cotton^24^, rice^36^, *moso bamboo*^37^ and *Populus*^38^. In addition, the majority of these AS events exhibited multiple AS types, which may be related to the ability of SMRT sequencing to read long sequences and thus to identify complex AS types^38^. Several studies have demonstrated that AS is a highly tissue-specific from of regulation^24,25,38^. To determine whether the AS in various tissues of tea plants also supports these conclusions, the AS events of the apical bud (Bud) and the second leaf (SL) were quantitatively identified and analyzed with RNA-seq data using the rMATS tools^39^. In total, 1,297 differential AS (DAS) events were identified, including 366 IR events (164 in Bud, 202 in SL), 526 ES events (252 in Bud, 274 in SL), 187 A3SS events (89 in Bud, 98 in SL), 132 A5SS events (60 in Bud, 72 in SL) and 106 MXE events (65 in Bud, 41 in SL) based on significant junctions and reads on targets (adjusted P-value < 0.01) (Fig. 2D). These results indicate that AS events occur extensively in tea plants, and the different splices have tissue specific expression.

### Functional annotation of transcripts

The 93,883 non-redundant transcripts were annotated using the best BLASTX hit from eight protein databases. In total, 30,164 transcripts were annotated in the COG database; 50,168 were annotated in the GO database; 34,655 were annotated in the KEGG database; 49,338 were annotated in the KOG database; 57,301 were annotated in Pfam; 55,860 were annotated in Swiss-Prot; 75,494 were annotated in eggNOG and 77400 were annotated in NR. Ultimately, 77,775 transcripts were annotated in the eight databases (Table 3).

**Table 3:** The statistical analysis of the annotated transcripts.

Databases	Annotated transcripts
COG	30,164
GO	50,168
KEGG	34,655
KOG	49,388
Pfam	57,301
Swiss-Prot	55,860
eggNOG	75,494
NR	77,400
All	77,775

**Figure 3:**
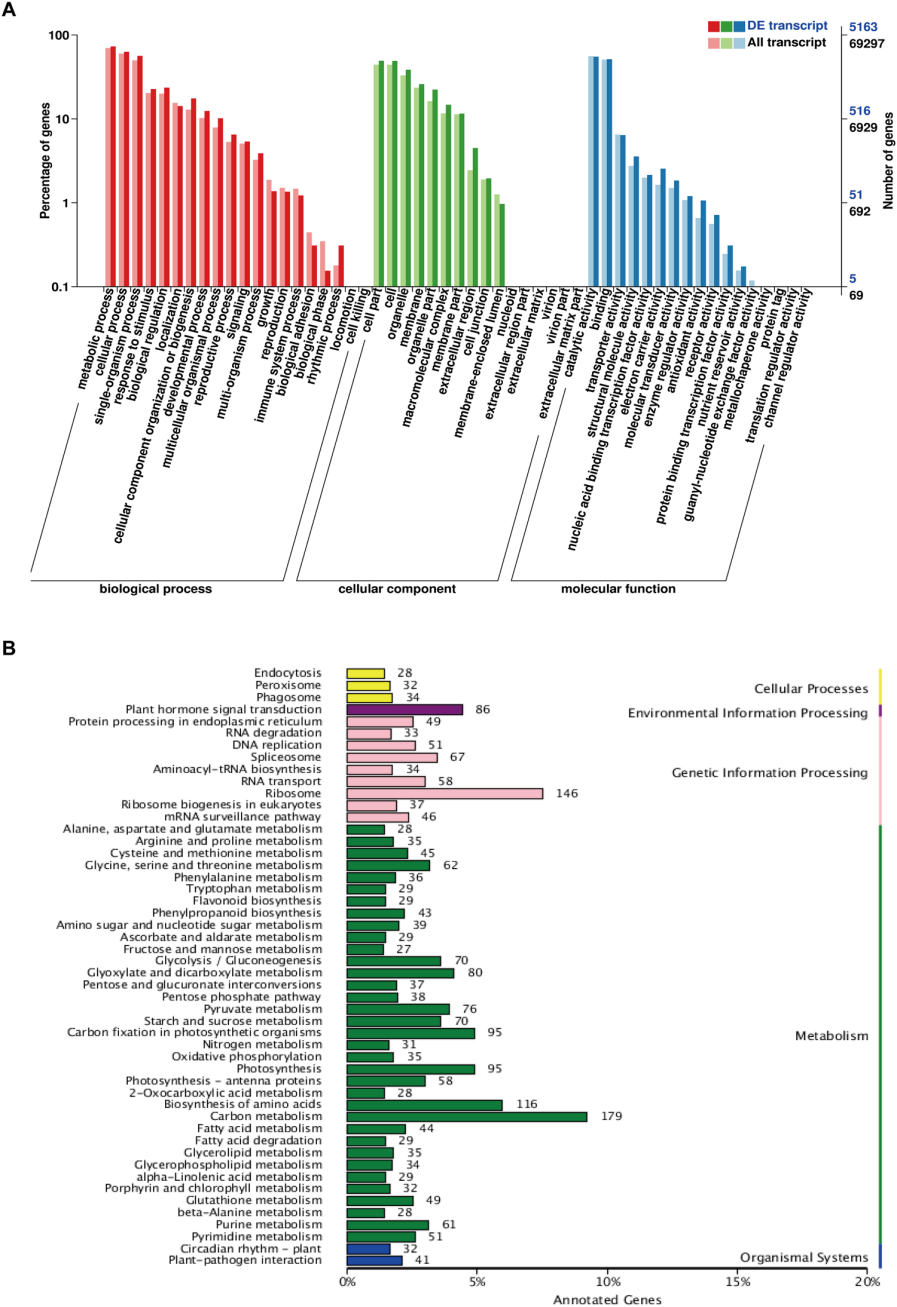
Functional classifications of the transcripts. (**A**) GO annotation classification statistics of the differential expression transcripts. (**B**) The KEGG pathway classification statistics of the differential expression transcripts.

To further functionally classify these transcripts, the GO terms analysis was performed. In total, 69,297 transcripts were assigned GO terms and 53 GO terms were enriched, which could be classified into three major categories (biological process, cellular component and molecular function) (Fig. 3A). In the biological process (BP) classification, the major subcategories were “metabolic process”, “cellular process” and “single-organism process”. For the cellular component (CC) classification, “cell part”, “cell” and “organelle part” were the three major subgroups. The “catalytic activity” and “binding” were the most dominant subcategories in the molecular function (MF) classification. The GO annotation classification of the DETs was consistent with that of all transcripts. Additionally, “general function prediction only”, “transcription” and “replication, recombination, and repair” were the three major subcategories in the COG functional classification analysis of the transcripts (Supplementary Fig. S4).

To better explore the function of these DETs, the DETs were then searched against the KEGG pathway database. A total of 122 KEGG pathways were identified, and the majority of them were metabolic related pathways, such as flavonoid biosynthesis (ko00941), alanine, aspartate and glutamate metabolism (ko00250), flavone and flavonol biosynthesis (ko00944), nitrogen metabolism (ko00910) and other secondary metabolic pathways of tea plants. Additionally, there were also 86 and 41 DETs involved in plant hormone signal transduction and plant-pathogen interaction pathways, respectively (Fig. 3B). These results suggested that these DETs were not only involved in the regulation of secondary metabolism of tea plants, but also in biological stress responses.

### Characterization of the transcripts in flavonoid biosynthesis

**Figure 4:**
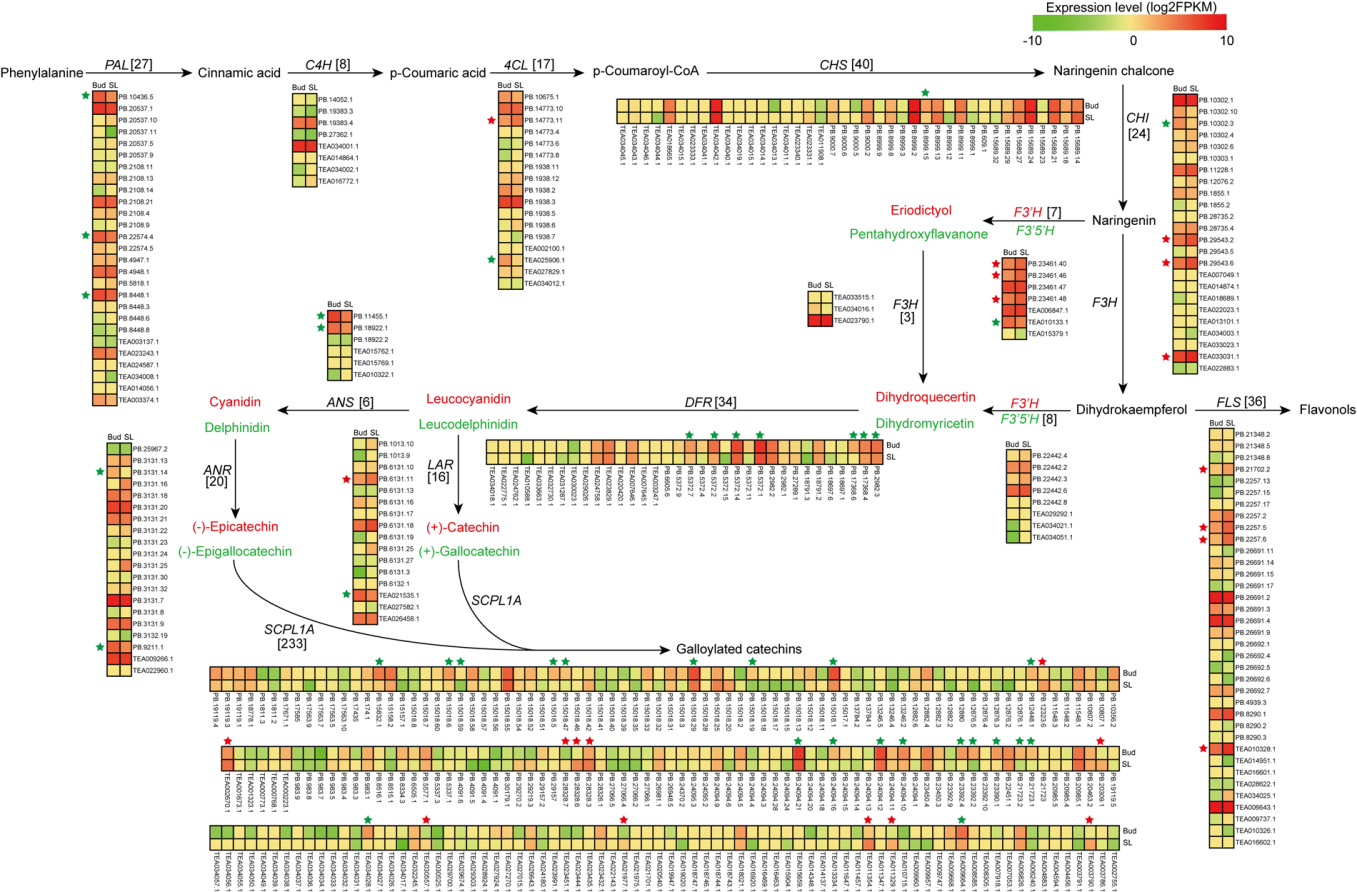
The flavonoid biosynthetic pathway in tea plants. The numbers in brackets following or under of each gene name indicate the number oftranscripts identified in this study. The expression levels of the transcripts in the two tissues are represented by heat maps. PAL, phenylalanine ammonia-lyase; C4H, cinnamic acid 4-hydroxylase; 4CL, 4-coumarate-CoAligase; CHS, chalcone synthase; CHI, chalcone isomerase; F3H, flavanone 3-hydroxylase; F3′H, flavonoid 3′-hydroxylase; F3′,5′H, flavonoid 3′,5′-hydroxylase; FLS, flavonol synthase; DFR, dihydroflavonol 4-reductase; ANS, anthocyanidin synthase; ANR, anthocyanidin reductase; LAR, leucocyanidin reductase; SCPL1A, type 1A serine carboxypeptidase-like acyltransferases.

Catechins are mainly synthesized through the phenylpropanoid pathway and the flavonoid pathway, and the key regulatory genes of these pathways have been reported^5,9^. In this study, based on the SMRT sequencing, a total of 512 transcripts were annotated in phenylpropanoid and flavonoid pathways (Supplementary Table S4). The expression levels of these transcripts in Bud and SL were calculated using FPKM values, from which 479 transcripts were detected and showed a varied expression patterns (Fig. 4). Additionally, many of the transcripts were identified as the different alternatively spliced isoforms from the same gene (Supplementary Table S5). For the phenylpropanoid pathway, twenty-seven, eight and seventeen transcripts were annotated as *PAL*, *C4H* and *4CL*, respectively. Among them, three transcripts (PB.10436.5, PB.22574.4 and PB.8448.1) of *PAL* and two transcripts (PB.14773.11 and TEA025906.1) of *4CL* were DETs in the two tissues. Moreover, PB.14773.11 was transcribed from TEA025906 by intron retention, which indicated that the tissue expression specificity for AS events. For the flavonoid pathway, the key genes, including *CHS* (40), *CHI* (24), *F3*′*H* (7), *F3H* (3), *F3*′*5*′*H* (8), *FLS* (36), *DFR* (34), *ANS* (6), *LAR* (16), *ANR* (20) and *SCPL* (233) were detected from the two tissues (Fig. 4). Among of them, one transcript of *CHS*, four of *CHI*, *F3*′*H* and *FLS*, seven of *DFR*, two of *ANS*, *LAR* and *ANR* and thirty-one of *SCPL* were DETs. Of the eleven key genes, AS events were identified for all of them except for *F3H*, *F3*′*5*′*H* and *ANS*, and multiple types of AS events for the same gene were also detected (Supplementary Table S5). For example, TEA023451 was one of the *SCPL1A* genes, which plays vital roles in the galloylation offlavan-3-ols^5^. In this study, ten AS events of TEA023451 were detected, including seven IR types, two A5SS types and one A3SS type (Supplementary Table S5). Certain types of AS events of *CHS*, *CHI*, *DFR*, *LAR* and *ANR* have been identified in previous studies, and some of them are prevalent and specific in certain tea plant cultivars^5,18^. Our results were consistent with previous studies, and supplemented the identification of AS events for key genes in the flavonoid pathway of tea plant. However, further research is warranted to investigate how these AS events regulate the synthesis of catechins in tea plants.

### Characterization of the transcripts in theanine biosynthesis

**Figure 5:**
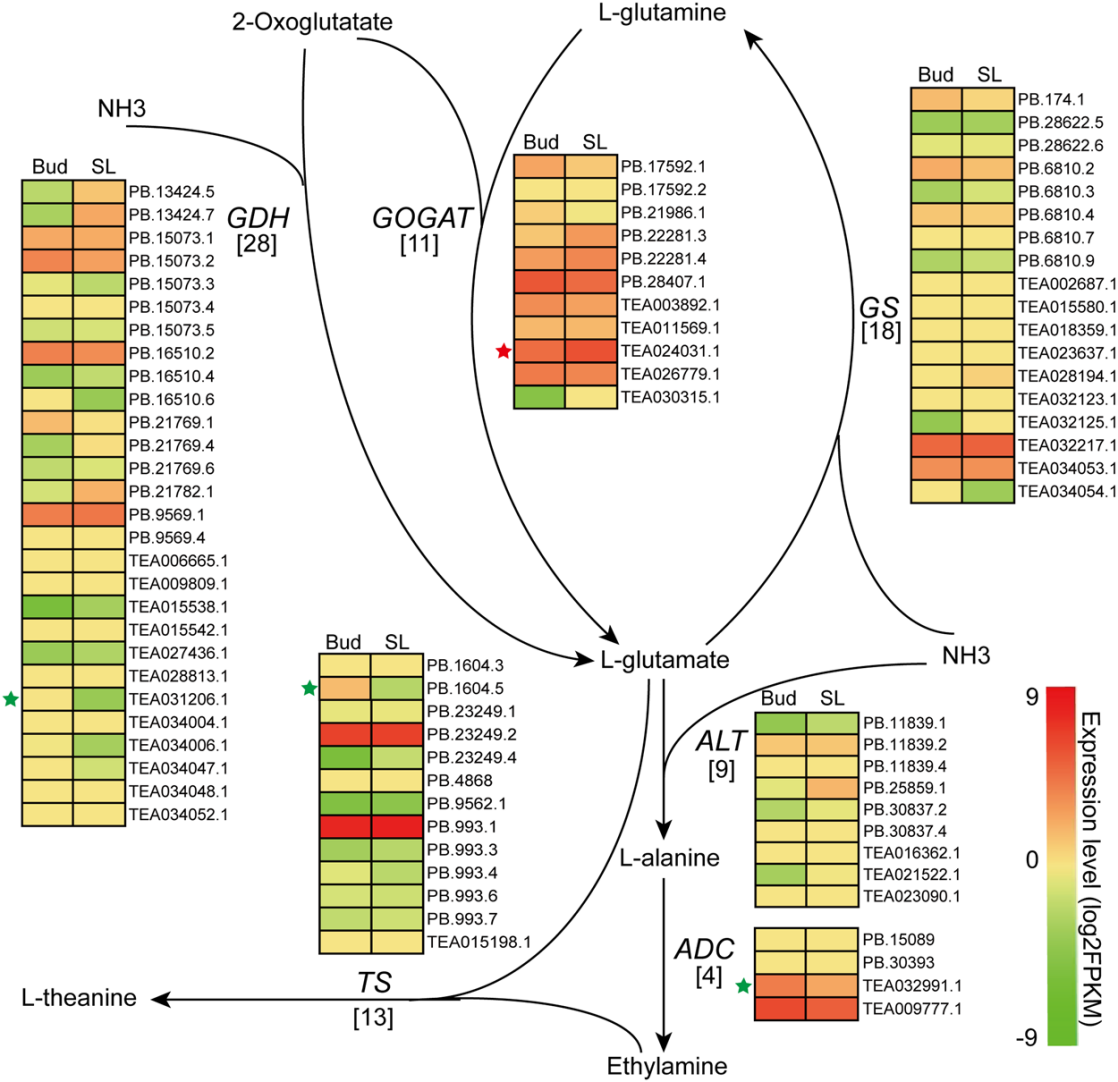
The theanine biosynthetic pathway in tea plants. The numbers in brackets under each gene name indicates the number oftranscripts identified in this study. The expression levels of the transcripts in the two tissues are represented by heat maps. GDH, glutamate dehydrogenase; GOGAT, glutamate synthase; GS, glutamine synthetase; ALT, alanine aminotransferase; ADC, argininedecarboxylase; TS, theanine synthetase.

Theanine is synthesized from glutamic acid and ethylamine and catalyzed by theanine synthetase (TS)^5^. In addition to *TS*, previous studies have shown that there are five additional key genes (*GDH, GOGAT, GS, ALT* and *ADC*) that also closely related to this process (Fig. 5). In this study, twenty-eight *GDH*s, eleven *GOGAT*s, eighteen *GS*s, nine *ALT*s, four *ADC*s and thirteen *TS*s were identified (Supplementary Table S6). The expression patterns of these genes in various tissues are shown in Fig. 5. Among of them, TEA031206.1 (*GDH*), TEA024031.1 (*GOGAT*), TEA032991.1 (*ADC*) and PB.1604.5 (*TS*) were differentially expressed between the two tissues. Additionally, AS events were also detected in *GDH*, *GS*, *ALT* and *TS* (Supplementary Table S5). *CsTSI* (TEA015198) is the only confirmed theanine synthase gene in tea plants at present^5^. In this study, PB.1604.2 and PB.1604.5 were identified as two AS isoforms of TEA015198, and PB.1604.5 was significantly down-regulated in SL, while the expression of PB.1604.2 was not detected based on the RNA-seq analysis, the expression patterns of them were also validated by RT-PCR (Fig. 7). According to the RT-PCR, PB.1604.5 was detected in two tissues, but PB.1604.2 was only detected in SL with a slight band (Fig. 7, Supplementary Fig. S6). This result indicated that PB.1604.5 may be the main functional transcriptional form of theanine synthase.

### Characterization of the transcripts in caffeine biosynthesis

**Figure 6:**
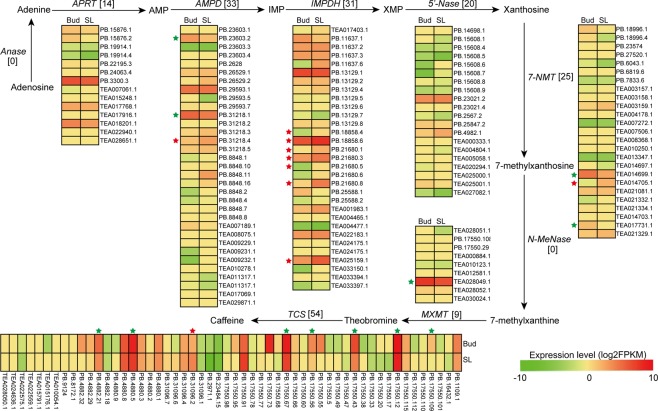
The caffeine biosynthetic pathway in tea plants.The numbers in brackets following or under each gene name indicate the number oftranscripts identified in this study. The expression levels of the transcripts in the two tissues are represented by heat maps. Anase, adenosine nucleosidase; APRT, adenine phosphoribosyltransferase; AMPD, AMP deaminase;IMPDH, IMP dehydrogenase; 5′-Nase, 5′-nucleotidase; 7-NMT, 7-methylxanthosine synthase; N-MeNase, N-methylnucleotidase; MXMT, theobromine synthase; TCS, tea caffeine synthase.

**Figure 7:**
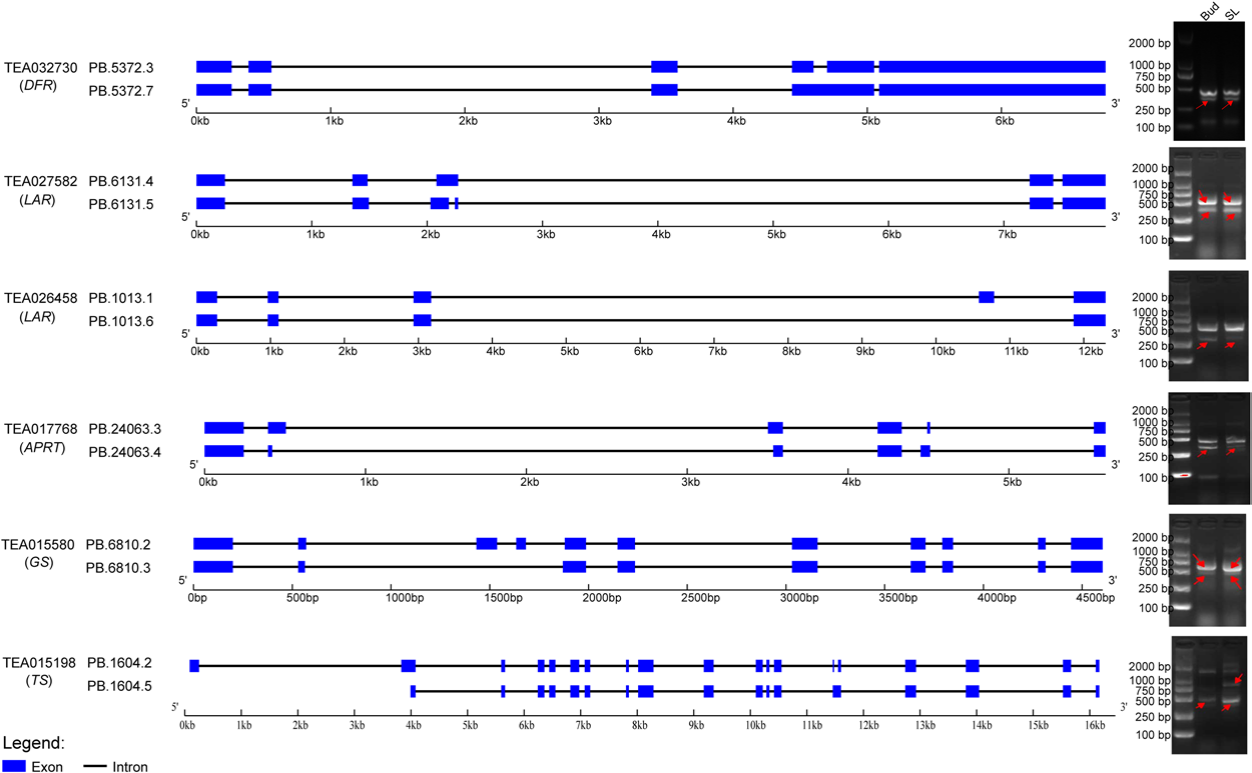
Validation of the differential AS (DAS) events using RT-PCR. The exon-intron structures of the genes were obtained using the online program GSDS 2.0 (http://gsds.cbi.pku.edu.cn/). Sequence alignment of the amplified fragments are shown in Supplementary Fig. S6.

Caffeine is 1,3,7-trimethylxanthine, and the major direct biosynthesis pathway of caffeine is from xanthosine to 7-methylxanthosine to 7-methylxanthine to theobromine (3,7-dimethylxanthine) to caffeine^16^. Additionally, the xanthosine is synthesized from adenosine via the purine biosynthesis pathway. Taken together, a total of nine key genes are involved in caffeine biosynthesis (Fig. 6, Supplementary Table S7). In this study, for the purine biosynthesis pathway, fourteen *APRT*s, thirty-three *AMPD*s, thirty-one *IMPDH*s and twenty *5*′*Nase*s were identified. All of these genes were shown to have unique expression profiles, and three transcripts of *APRT* and seven transcripts of *IMPDH* were DETs in the two tissues (Fig. 6). Moreover, AS events of the four genes were also detected, and different AS events from the same gene were shown to have different expression patterns (Supplementary Table S5). For example, PB.23603.2 and PB.23603.4 were the two transcripts of *AMPD* (TEA009231) and both were identified as IR events. The former was significantly down-regulated in SL, while the expression level of the latter did not differ between Bud and SL. For the purine modification steps, twenty-five transcripts of *NMT*, nine of *MXMT* and fifty-four of *TCS* were identified. Among them, there were three, one and eight that were DETs. The AS events of them were also identified from *NMT*s and *TCS*s. For example, PB.4880.1 and PB.4880.2 were characterized as an IR event of TEA028050 (*TCS*), although there was no significant difference in their expression levels in both tissues, the expression level of the former was higher than that of the latter (Figs 6, 7).

### Correlation analysis of gene expression and secondary metabolite accumulation

To further explore the relationship between AS and the regulation of secondary metabolisms, the correlation analysis between the expression of the secondary metabolism-related structural genes with AS transcripts identified in this study (Supplementary Table S5) and the accumulation of secondary metabolites was performed. Based on the Gene Expression and Metabolite accumulation Correlation Analysis Tool (http://tpia.teaplant.org/Gene2Metabolite.html) in the Tea Plant Information Archive (http://tpia.teaplant.org/index.html), a Pearson’s correlation analysis was performed using the expression data of the 16 closely representative *Camellia* species from Section Thea. More than 68% of the selected genes (48/70) showed significant correlations (P < 0.05) between the gene expression and the accumulation of at least one of secondary metabolites (Supplementary Fig. S5, Supplementary Table S9), which indicated that the changes in secondary metabolites were regulated by the mRNA expression of secondary metabolism-related structural genes. In our study, at least one AS transcript was identified from these genes (Supplementary Table S5), and the different isoforms of the same gene also exhibited various expression patterns in Bud and SL (Figs 4, 5, 6). These phenomena had been observed in tea plant. For example, the AS transcripts but not the full-length transcripts of *CsMYB* and *CsPAL* were highly correlated with the accumulation of epicatechin-3-gallate^40^, which suggested that AS influenced the biosynthesis of the secondary metabolites.

The tea plant genome (*Camellia sinensis* var. sinensis) was shown to be approximately 3.1 Gb and to contain two rounds of whole-genome duplications (WGDs), which led to many repeated sequences in the tea plant genome^5^. The copy numbers of secondary metabolism-related genes were also increased. For example, there are twenty-two *SCPL* genes in the tea plant, which is twice the number of *SCPL* genes in grapes^5^. In our study, 233 transcripts of *SCPL* were identified, and different expression patterns of these transcripts were also observed, which revealed that the multigene family members of the genes involved in the secondary metabolism of tea plant greatly limited our understanding of the regulatory mechanisms. Additionally, AS could also significantly increase the diversity and complexity of the transcriptome^21,22^. In tea plants, some of the AS events had been identified from certain functional genes, and the differently spliced isoforms of the same gene showed different effects on subcellular localization, gene expression and stress response^7,11,33,41^. However, due to the lack of a reference genome, it was difficult to comprehensively identify the AS in tea plants. Although the AS events of the secondary metabolism-related genes in tea plants had been identified by Xu *et al*. using single-molecule sequencing approach and BAC library, the AS events were only identified from eight genes involved in the biosynthesis of flavonoids, theanine and caffeine^7^. Then based on RNA-seq and Iso-Seq, a large number of AS events were identified in gene transcripts of the flavonoid biosynthesis pathway by the same research group, and they also confirmed that certain AS isoforms, rather than the full-length transcripts, were the major transcripts involved in this pathway and were correlated with the catechin content in the tea plant^40^. In our study, the AS events in tea plants were fully identified using the latest reference genome of tea plants combined with the single-molecule sequencing and RNA-seq technologies. The results revealed that most of the key genes involved in the three major secondary metabolic pathways of the tea plant underwent AS, and the different spliced isoforms exhibited tissue specific expression patterns. Our results not only complemented the previous research, but also deepened our understanding of the effects of AS on the regulation of secondary metabolism in tea plants. However, the authenticity and function of these AS events warrant further clarification.

### Identification of fusion transcripts and long non-coding RNAs

Fusion transcripts, which are also known as chimeric transcripts, are caused by somatic chromosomal rearrangement and are the common factor in the development of cancers in humans^42^. The study of fusion transcripts in plants is limited at present. In this study, 5,777 transcripts were identified as fusion transcripts using the pbtranscript-ToFU package (Supplementary Table S8).

**Figure 8:**
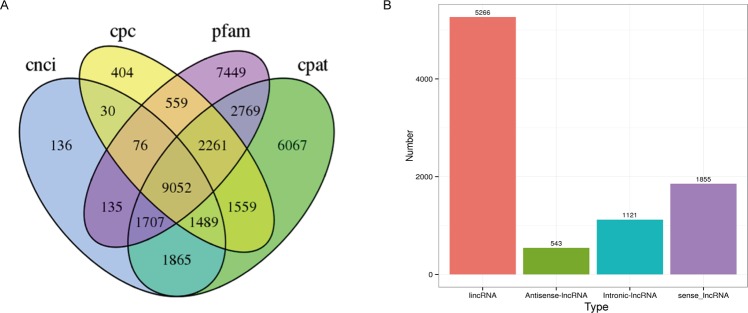
The number of predicted lncRNAs. (**A**) Venn diagram of the number of predicted lncRNAs according to CNCI, CPC, Pfam and CPAT analysis. (**B**) The distribution of different types of lncRNAs.

Long non-coding RNAs (lncRNAs) are widespread in eukaryotic genomes, and play crucial roles in varied biological processes^43,44^. Based on their genomic origins, lncRNAs can be placed into the following four categories: lncRNA in intergenic (lincRNA), lncRNA in intronic (intronic-lncRNA), and lncRNA coding regions in the sense (sense-lncRNA) and antisense (antisense-lncRNA) directions^44^. Here, a total of 9,052 lncRNAs were predicted by CNCI, CPC, Pfam and CPAT analysis (Fig. 8A), and the number of each type of lncRNA is shown in Fig. 8B. The functions of the fusion transcripts and lncRNAs identified in this study will require further investigation in the future.

## Conclusions

In conclusion, in this study, RNA-seq and full-length transcriptome techniques were used to explore the regulation of alternative splicing events in the secondary metabolism pathways in tea plants. A total of 93,883 transcripts were identified, including many alternatively spliced isoforms. Meanwhile, 1,297 differential AS events were detected between the apical bud and the second leaf under the apical bud, suggesting that AS events not only occer extensively in tea plants but are tissue-specific. The expression patterns of the transcripts involved in flavonoid, theanine and caffeine biosynthesis were characterized, and it was shown that the differential alternatively spliced isoforms of the same gene played different roles in the regulation of secondary metabolism during the tea plant development and growth. Additionally, 5,777 fusion transcripts and 9,052 lncRNAs were also predicted in this study, which likely play a special role in the regulation of secondary metabolism of the tea plant. Our results expand the knowledge of the mechanisms underlying the regulation of secondary metabolism in tea plants and provide a valuable resource for future studies into the role of AS in flavonoid, theanine and caffeine metabolism in tea plants.

## Materials and Methods

### Plant materials and RNA preparation

Eight-year-old tea plants (*Camellia sinensis* var. sinensis cv. Qiancha 8) were planted in the Germplasm Tea Repository of Guizhou Tea Research Institute located at Guiyang (N26°30′20″, E106°39′26″), Guizhou Province, China. The samples (apical bud, first leaf, second leaf, and roots) were collected from the same tea plant (Supplementary Fig. S1). Among them, the apical bud, first leaf and second leaf were collected on April 2, 2018, and roots were collected on March 12, 2018. All samples were immediately frozen in liquid nitrogen and stored in a −80 °C refrigerator. The total RNAs were extracted using the TaKaRa MiniBEST Plant RNA Extraction Kit (TaKaRa) with DNase I treatment toremove genomic DNA. The concentration and quality of the total RNAs were assessed using an ND-1000 spectrophotometer (NanoDrop Technologies) and an Agilent 2100 Bioanalyzer (Agilent Technologies).

### PacBio library construction and sequencing

The total RNAs from the four different tissues were mixed equally for the PacBio library construction. The library was prepared according to the Isoform Sequencing (Iso-Seq) protocol, as described by PacificBiosciences. The first-strand cDNA was synthesized using the Clontech SMARTerTM PCR cDNA synthesis Kit (Clontech). After the PCR amplification, the products were purified with AMPurePB magnetic beads. The concentration and size of the purified products were detected using the Qubit 2.0 Fluorometer (Life Technologies) and Agilent 2100 Bioanalyzer (Agilent Technologies). Then, the size-selected cDNAs were amplified and used to construct the SMRTbell Template libraries using the SMRTBell Template Prep Kit according to the manufacturer’s instruction. The quality of the libraries was assessed using the Qubit 2.0 Fluorometer (Life Technologies) and the Agilent 2100 Bioanalyzer (Agilent Technologies). Finally, a total of three SMRT cells were sequenced on the PacBio Sequel platform by Biomarker Tech (Beijing China).

### Illumina RNA-seq library construction and sequencing

The total RNAs of the apical bud (Bud-1, -2, -3) and the second leaf (SL-1, -2, -3) were used to construct the RNA-seq libraries. The sequencing libraries were generated using the NEBNext UltraTM RNA Library Prep Kit for Illumina (NEB) according to the manufacturer’s instructions. In brief, the mRNA was enriched by oligo(dT) magnetic beads and was then cut into short fragments using the NEBNext First Strand SynthesisReaction Buffer (NEB). The first strand cDNA was synthesized using the Random hexamerprimers and M-MuLV Reverse Transcriptase and both DNA polymeraseI and RNase H were used to synthesize the second strand cDNA. After the adenylation of 3′ ends of the DNA fragments, hybridization was performed for the NEBNext Adaptor (NEB), which contained a hairpinloop structure. The AMPureXP system (Beckman Coulter) was used to size-select the cDNAs. The size selected cDNAs were incubated with the USER Enzyme (NEB) and were then amplified using Phusion High-Fidelity DNA polymerase and primers. The quality of the libraries was assessed using the Agilent 2100 Bioanalyzer (Agilent Technologies). The RNA-seq was performed on the Illumina Hiseq X Ten platform based on the Paired-End 150 (PE150) strategy by Biomarker Tech (Beijing China).

### PacBio long read processing

Raw reads were processed into error corrected reads of insert (ROIs) using the Iso-seq pipeline with minFullPass = 0 and minPredictedAccuracy = 0.80. Then the full-length, non-chemiric (FLNC) transcripts were determined by searching for the polyA tail signal and the 5′ and 3′ cDNA primers in the ROIs. Iterative Clustering for Error Correction (ICE) was used to obtain consensus isoforms, and the FL consensus sequences from ICE were polished using Quiver. The low quality transcripts were further corrected with the RNA-seq data. High quality FL transcripts were classified with the criteria of post-correction accuracy above 99%. Finally, the FL consensus sequences were mapped to the tea plant reference genome (http://pcsb.ahau.edu.cn:8080/CSS/) using Genomic Mapping and Alignment Program (GMAP)^45^. The mapped reads were further collapsed by pbtranscript-ToFU package with min-coverage = 85% and min-identity = 90%. The 5′ difference reads were not considered when collapsing redundant transcripts. The BUSCO^34^ was used to evaluate the integrity of the transcriptome without redundancy, and the number of embryophyta gene sets used in this evaluation was 1,440.

### RNA-seq data analysis

First, raw reads were processed through in-house Perl scripts. In this step, clean reads were obtained by removing reads containing the adapter, reads containing unknown bases (>10%) and low quality reads (when the percentage of low quality bases was over 50% in a read) from raw reads. At the same time, the Q20, Q30, GC-content and sequence duplication level of the clean reads were calculated. The clean reads were then mapped to the tea plant reference genome (http://pcsb.ahau.edu.cn:8080/CSS/) using Tophat2. Only those reads with a perfect match or one mismatch were further analyzed and annotated based on the reference genome. Fragments Per Kilobase of transcript per Million fragments mapped (FPKM) was used in calculating the expression level of genes or transcripts. Differential expression analysis between the two tissues was performed using the DESeq R package (1.10.1)^46^. The resulting P values were adjusted using the Benjamini and Hochberg’s approach for controlling for the false discovery rate (FDR). Genes with an adjusted P-value < 0.05, FDR ≤ 0.01 and fold change (FC) ≥ 2 (|log2 (fold change)| ≥ 1) identified by DESeq were assigned as differentially expressed.

### Identification of fusion transcripts from PacBio sequences

The pbtranscript-ToFU package was used to identify fusion transcripts. The transcripts that were screened as candidate fusion transcripts were mapped to two or more loci, the minimum coverage for each loci was at last 5%, the minimum coverage in bp was more than 1 bp, the total coverage was at last 95% and the distance between the loci was at least 10 kb. The Illumina short reads generated from the HiSeq X Ten platform were further used to validate candidate fusion transcripts.

### Identification of alternative splicing events

The alternative splicing (AS) events, including intron retention (IR), alternative 5′ splice site (A5SS), alternative 3′ splice site (A3SS), exon skipping (ES)and mutually exclusive exon (MEE) were identified by the AStalavista tool^35^. The differential alternative splicing (DAS) events were detected using the rMATS tools^39^ based on the RNA-seq data.

### Identification of lncRNAs from PacBio sequences

Four computational approaches, including coding potential calculator (CPC), coding-non-coding index (CNCI), coding potential assessment tool (CPAT) and the Pfam database were combined to sort non-protein coding RNA candidates from putative protein-coding RNAs in the transcripts. The putative protein-coding RNAs were filtered out using a minimum length and exon number threshold. The transcripts with lengths greater than 200 nt and more than two exons were selected as lncRNA candidates. The putative lncRNAs were further screened using CPC, CNCI, CPAT and Pfam, and the common results obtained by these four approaches were used for the subsequent analysis.

### Functional annotation of transcripts

The non-redundant transcripts were annotated based on the best BLASTX hit following the eight public databases, including NR (NCBI non-redundant protein sequences), Pfam (Protein family), KOG (euKaryotic Ortholog Groups), COG (Clusters of Orthologous Groups of proteins), eggNOG (evolutionary genealogy of genes: Non-supervised Orthologous Groups), Swiss-Prot (A manually annotated and reviewed protein sequence database), KEGG (Kyoto Encyclopedia of Genes and Genomes) and GO (Gene Ontology). All of the searches were performed with the threshold of E-value ≤ 10-5. A GO enrichment analysis was implemented using the GOseq R packages based Wallenius non-central hyper-geometric distribution^47^. A KEGG pathways enrichment analysis was performed using the KOBAS software^48,49^.

### Correlation analysis of gene expression and secondary metabolite accumulation

The correlation analysis was performed using the Gene Expression and Metabolite accumulation Correlation Analysis Tool (http://tpia.teaplant.org/Gene2Metabolite.html) in the Tea Plant Information Archive (TPIA, http://tpia.teaplant.org/index.html). TPIA is the most comprehensive public database of tea plant genome at present. The secondary metabolism-related structural genes with AS transcripts identified in this study were selected as target genes, and the expression patterns of these genes among leaves of 16 closely representative *Camellia* species from Section Thea (http://tpia.teaplant.org/Correlationanalysis.html) were used to explore the relationship between gene expression and secondary metabolite accumulation. Three methods including Pearson, Spearman and Kendall were used for the correlation assessment, and the data filtered with |R| > 0.5 and P-value < 0.05.

### Quantitative real-time PCR (qPCR) and reverse transcription PCR (RT-PCR) analysis

For the qPCR and RT-PCR analysis, the reverse transcription was performed using the PrimeScriptTM RTreagent Kit with gDNA Eraser (TaKaRa) according to the manufacturer’s instructions. The gene or transcript specific primers were designed with Primer Premier 5 and synthesized by Sangon Biotech (Shanghai China). The qPCR reactions were performed with the ABI Vii ATM 7Real-time PCR System using the GoTaq® qPCR Master Mix (Promega). The *CsTBP* gene was selected as a reference gene^50^. The relative expression level was calculated using the following formula: FC = 2^−△△CT^ ^38^. The RT-PCR reactions were performed in a BIO-RAD C1000 PCR System, and the PCR amplifications were performed in 20 μl reaction mixtures, which contained 10 μl of Premix Taq (TaKaRa), 1 μl of template cDNA, 1 μl of each primer and 7 µl of sterile double distilled water. The PCR products were examined on 2.0% agarose gels. The primers used in this study are listed in Supplementary Table S10.

## Supplementary information

Supplementary Figures

Supplementary Tables

## Data Availability

The raw data have been deposited in the Genome Sequence Archive^51^ in BIG Data Center, Beijing Institute of Genomics (BIG), Chinese Academy of Sciences, under accession number CRA001188 that is publicly accessible at http://bigd.big.ac.cn/gsa. The full-length transcripts information (in fasta file and GFF fire) by PacBio Iso-Seq in this study were deposited into the figshare website: 10.6084/m9.figshare.7403933.
