# Supplementary material for: Comprehensive identification of the full-length transcripts and alternative splicing related to the secondary metabolism pathways in the tea plant (Camellia sinensis)
Source: Sci Rep. 2019 Feb 25;9:2709. doi: 10.1038/s41598-019-39286-z (PMC6389920; doi:10.1038/s41598-019-39286-z)
Supplement: Supplementary file 1 — Supplementary Figures [file 41598_2019_39286_MOESM1_ESM.pdf]

Comprehensive identification of the full-length transcripts and alternative splicing related to the secondary metabolism pathways in the tea plant (*Camellia sinensis*)

Dahe Qiao[1], Chun Yang[1], Juan Chen[1], Yan Guo[1], Yan Li[1], Suzhen Niu[2], Kemei Cao[2] & Zhengwu Chen[1]*

[1] Tea Research Institute, Guizhou Academy of Agricultural Science, Guiyang 550006 Guizhou, China. [2] College of tea science, Guizhou University, Guiyang 550025 Guizhou, China

*Correspondence: Z.W.C, Email: zwchentea@163.com, Tel.: +86- 0851-83761972

Additional Information

Figure. S1 Flow diagram of the experimental design and joint analysis for PacBio sequencing and Illumina sequencing.

Figure. S2 The results of transcriptome integrity assessment based on BUSCO using an embryophyta gene set. The total number of embryophyta gene sets used in this evaluation was 1,440.

Figure. S3 Differential expression analysis of transcripts. (A) Correlation analysis of the expression between two samples. (B) The MA plot of the differentially expressed transcripts. Each point represents a transcript. The x-axis represents A value, which is the logarithm of the mean FPKM values of the two samples, and the y-axis represents M value, which is the logarithm of fold change (FC) in gene expression between two samples. The green dots and red dots represent down and up-regulated differentially expressed transcripts, respectively, and the black dots represent non-differentially expressed transcripts.

Figure. S4 Functional distribution of COG annotation of the transcripts.

Figure. S5 Correlation analysis of gene expression and secondary metabolite accumulation. The secondary metabolism-related structural genes with AS transcripts identified in this study were selected and the correlation analysis were performed on using the Gene Expression and Metabolite accumulation Correlation Analysis Tool (http://tpia.teaplant.org/Gene2Metabolite.html).

Figure. S6 Sequence alignment of the amplified fragments of the AS isoforms. The primer sequences were underlined in red.

Table S1. Comparison of single molecule sequencing data of tea plant between two PacBio sequencing platforms.

Table S2. The differential expression transcripts identified in this study.

Table S3. The character of AS events identified in this study.

Table S4. The transcripts involved in flavonoid biosynthesis.

Table S5. The AS events of the transcripts involved in flavonoid, theanine and caffeine biosynthesis.

Table S6. The transcripts involved in theanine biosynthesis.

Table S7. The transcripts involved in caffeine biosynthesis.

Table S8. The character of fusion transcripts identified in this study.

Table S9. Correlation analysis of the gene expression and secondary metabolite accumulation.

Table S10. The primers used in this study.

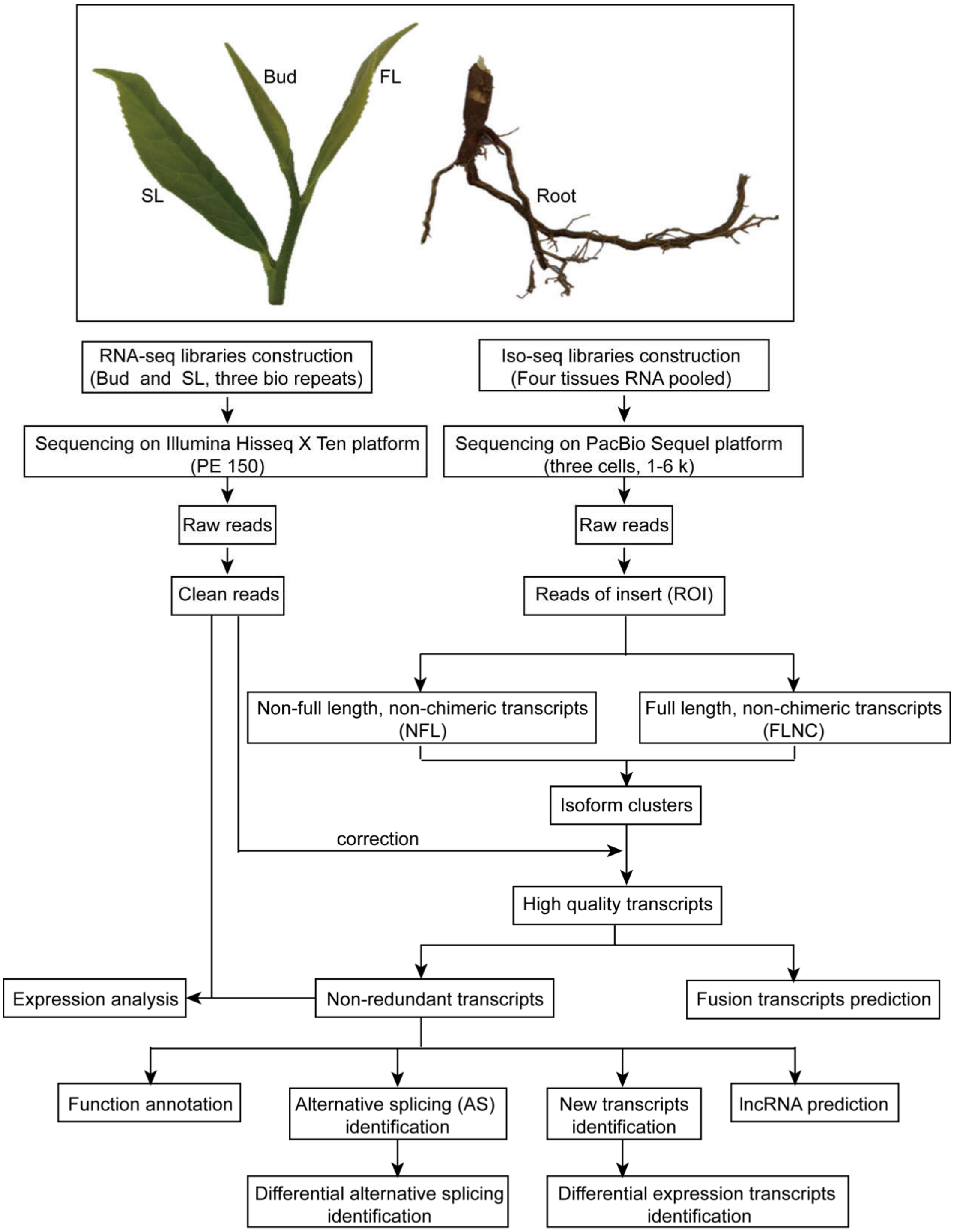

Figure S1. Flow diagram of the experimental design and joint analysis for PacBio sequencing and Illumina sequencing.

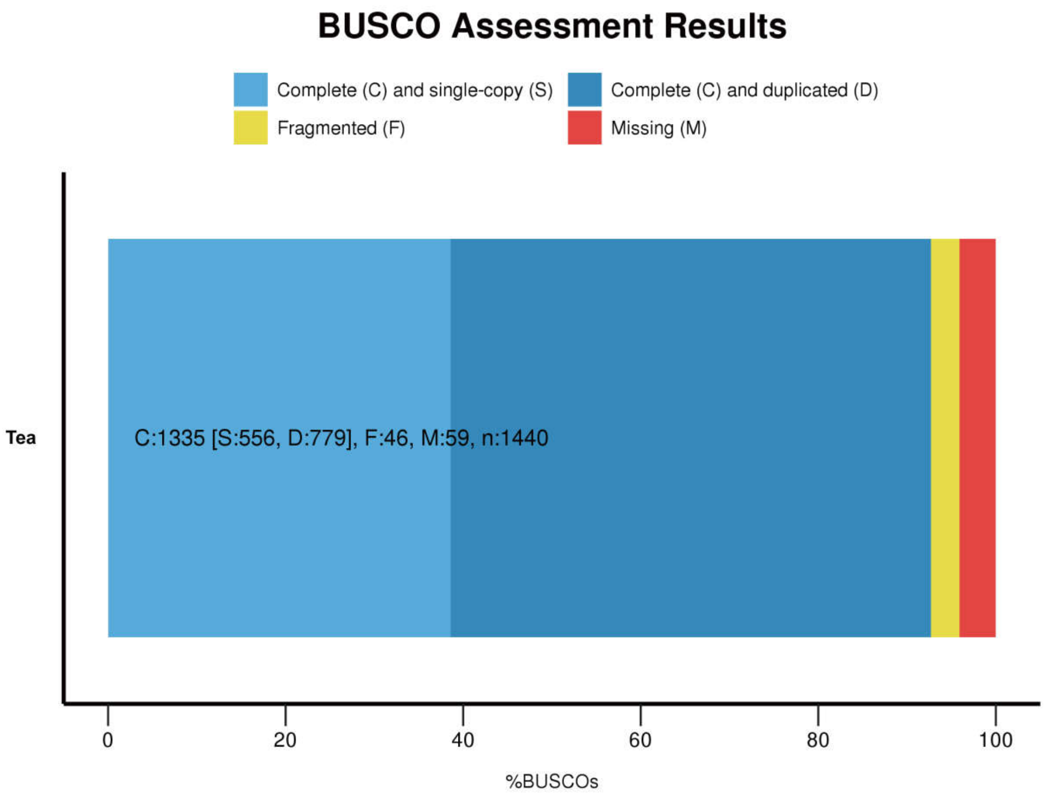

Figure S2. The results of transcriptome integrity assessment based on BUSCO using an embryophyta gene set. The total number of embryophyta gene sets used in this evaluation was 1,440.

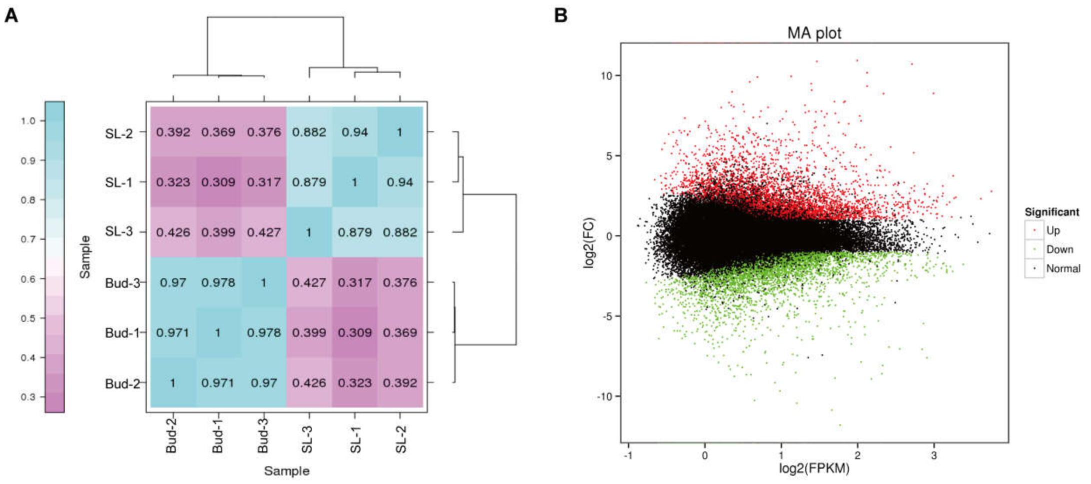

Figure S3. Differential expression analysis of transcripts. (A) Correlation analysis of the expression between two samples. (B) The MA plot of the differentially expressed transcripts. Each point represents a transcript. The x-axis represents A value, which is the logarithm of the mean FPKM values of the two samples, and the y-axis represents M value, which is the logarithm of fold change (FC) in gene expression between two samples. The green dots and red dots represent down and up-regulated differentially expressed transcripts, respectively, and the black dots represent non-differentially expressed transcripts.

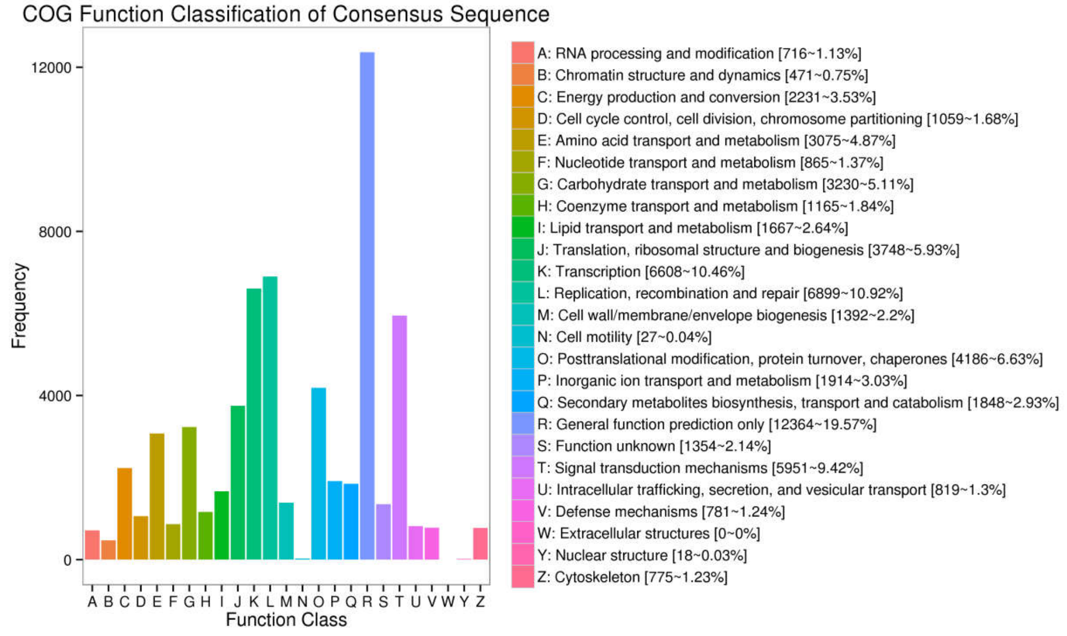

Figure S4. Functional distribution of COG annotation of the transcripts.

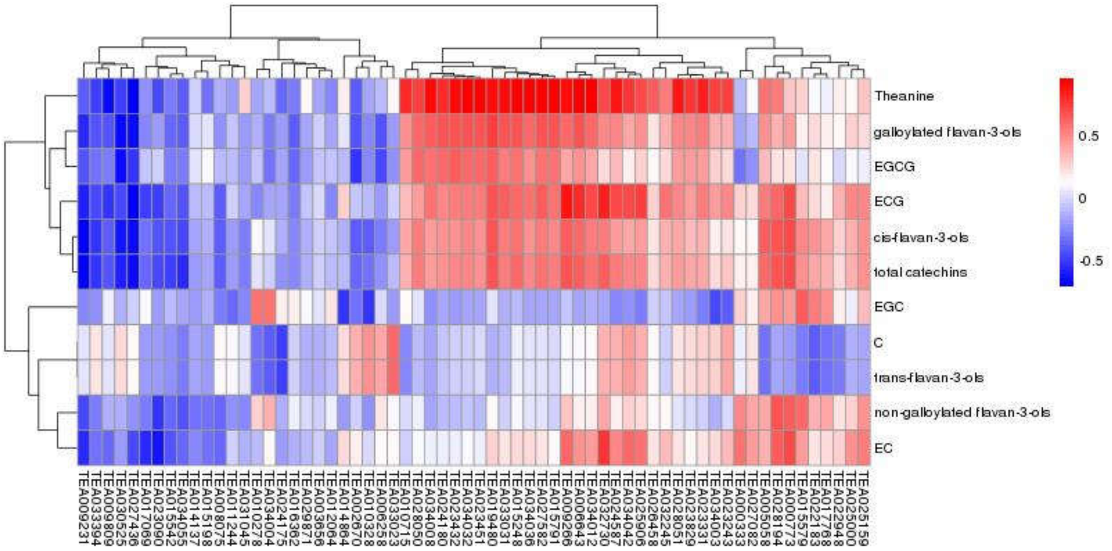

Figure S5. Correlation analysis of gene expression and secondary metabolite accumulation. The secondary metabolism-related structural genes with AS transcripts identified in this study were selected and the correlation analysis were performed on using the Gene Expression and Metabolite accumulation Correlation Analysis Tool (http://tpia.teaplant.org/Gene2Metabolite.html).

Figure S6. Sequence alignment of the amplified fragments of the AS isoforms. The primer sequences were underlined in red.

(A) Seq_0:PB.1604.5, Seq_1:PB.1604.2

```
Seq_0 -------------------------------------------------------------------------------- 0

Seq_1 GGTGTGGAGGTAGTAGACGCACACGCTCACAATCTGGTGGCTCTGGACTCCACTCTTCCTTTCCTCCAATGCTTTTCTGA 80


Seq_0 -------------------------------------------------------------------------------- 0

Seq_1 AGCCTACGGCGATGCTTTATTGCTCGCACCCCACGCTCTCAACTTCAAGAGAGGTATAAGGGATATTGCTGAACTGTATG 160


Seq_0 -------------------------------------------------------------------------------- 0

Seq_1 GATCTGAGTTATCCTTGGATGGCATTCAAAAATACCGCAAGGGCAATGGATTGCAATCCATAAGCTCAATATGCTTCAAG 240


Seq_0 -------------------------------------------------------------------------------- 0

Seq_1 GCTGCAAGAATCGCTGCAATACTCATTGATGATGGAATTGAGTTTGACAAAATGCATGACATTGAATGGCATAGGAATTT 320


Seq_0 -------------------------------------------------------------------------------- 0

Seq_1 TGCACCGGTGGTTGGTAGAATATTGAGAATTGAGCATCTGGCTGAGAAGATTCTTGATGAAGGGAGGCCAGATGGATCTA 400


Seq_0 ------------------TCACTGAAACATTTATCGGAAAGTTGAAGTCAGTTGCTAATAAAATTGTTGGCTTGAAAAGC 62

Seq_1 CCTGGACATTGGACAGTTTCACTGAAACATTTATCGGAAAGTTGAAGTCAGTTGCTAATAAAATTGTTGGCTTGAAAAGC 480


Seq_0 ATAGCTGCATACCGCAGTGGTCTTGAGATTAATACAAATGTCACAAGGAAGGAGGCTCAAGCGGGTCTTGTTGAAGTTTT 142

Seq_1 ATAGCTGCATACCGCAGTGGTCTTGAGATTAATACAAATGTCACAAGGAAGGAGGCTCAAGCAGGTCTTGTTGAAGTTTT 560


Seq_0 AAATGCTGGGAGCCCCGTTCGTATCACAAATAAAAACTTCATTGACTATCTCTTCGTGCAGAGTTTGGAGGTTGCCATAC 222

Seq_1 AAATGCTGGGAGCCCCGTTCGTATCACAAATAAAAACTTCATTGACTATCTCTTCGTGCAGAGTTTGGAGGTTGCCATAC 640


Seq_0 AATATGATTTGCCAATGCAGATACACACTGGTTTTGGAGATAAAGATTTGGATTTAAGGCTCTCCAATCCCTTGCATCTC 302

Seq_1 AATATGATTTGCCAATGCAGATACACACTGGTTTTGGAGATAAAGATTTGGATTTAAGGCTCTCCAATCCCTTGCATCTC 720


Seq_0 CGCACCCTTCTTGAGGACAAGAGATTCTCTAAGTGCCGCTTAGTACTTTTACATGCATCATACCCATTTTCAAAGGAAGC 382

Seq_1 CGCACCCTTCTTGAGGACAAGAGATTCTCTAAGTGCCGCTTAGTACTTTTACATGCATCATACCCATTTTCAAAGGAAGC 800


Seq_0 ATCATATCTAGCCTCCATTTATTCTCAGGTTTACCTTGATTTTGGTTTGGCTGTTC 438

Seq_1 ATCATATCTAGCCTCCATTTATTCTCAGGTTTACCTTGATTTTGGTTTGGCTGTTC 856
```

(B) Seq_0:PB.6131.4, Seq_1:PB.6131.5

```
Seq_0  GCGATTGATAGAAGAGTGTGGGGTGCCCTACACTTATATCTGCTGCAACTCTATTGCTTCTTGGCCATACTATGACAATA 80
Seq_1  GCGATTGATAGAAGAGTGTGGGGTGCCCTACACTTATATCTGCTGCAACTCTATTGCTTCTTGGCCA------------- 67

Seq_0  CTCACCCCTCGGAAGTAATTCCCCCTTTGGATGAGTTCCAAATTTATGGTGATGGCAGTGTCAAAGCATATTTTGTTGCA 160
Seq_1  ----------------------------------------AATTTATGGTGATGGCAGTGTCAAAGCATATTTTGTTGCA 107

Seq_0  GGCTCTGATATAGGAAAATTCACCATCAAAACCGTCGACGACATTCGTACGCTGAACAAATCGGTTCATTTCCGACCATC 240
Seq_1  GGCTCTGATATAGGAAAATTCACCATCAAAACCGTCGACGACATTCGTACGCTGAACAAATCGGTTCATTTCCGACCATC 187

Seq_0  CTGCAATTTTCTCAACATAAATGAGCTTGCATCTTTGTGGGAGAAGAAGATTGGACGCACTCTCCCCCGAGTCACTGTCT 320
Seq_1  CTGCAATTTTCTCAACATAAATGAGCTTGCATCTTTGTGGGAGAAGAAGATTGGACGCACTCTCCCCCGAGTCACTGTCT 267

Seq_0  CAGAAAATGACCTACTAGCAGCAGCAGCAGTGAATATAATCCCACAAAGTGTCGTTGCATCGTTCACACACGACATTTTC 400
Seq_1  CAGAAAATGACCTACTAGCAGCAGCAGCAGTGAATATAATCCCACAAAGTGTCGTTGCATCGTTCACACACGACATTTTC 347

Seq_0  ATTAAGGGATGCCAGATTAATTTTTCAATTGAAGGTCCTAATGACGTTGAAGTATGCAGCCTCTACCCTGATGAATCGTT 480
Seq_1  ATTAAGGGATGCCAGATTAATTTTTCAATTGAAGGTCCTAATGACGTTGAAGTATGCAGCCTCTACCCTGATGAATCGTT 427

Seq_0  CAGGACCGTTGATGAATGTTTCGATGATTTTGTTGTGAAGATGAATGGGAAGAATTTTACAGATGAGACGGATGG 555
Seq_1  CAGGACCGTTGATGAATGTTTCGATGATTTTGTTGTGAAGATGAATGGGAAGAATTTTACAGATGAGACGGATGG 502
```

(C) Seq_0:PB.1013.1, Seq_1:PB.1013.6

```
Seq_0  GATAGAGGAGTCAGGGGTGCCCTACACTTATATATGCTGCAACTCCATTGCTTCTTGGCCCTACTATGATAACACCCACC 80
Seq_1  GATAGAGGAGTCAGGGGTGCCCTACACTTATATATGCTGCAACTCCATTGCTTCTTGGCCCTACTATGATAACACCCACC 80

Seq_0  CTTCTGAGGTCATTCCTCCTTTGGATCGCTTCCAAATTTATGGTGATGGCACTGTCAAAGCTTACTTTGTTGATGGGTCT 160
Seq_1  CTTCTGAGGTCATTCCTCCTTTGGATCGCTTCCAAATTTATGGTGATGGCACTGTCAAAG-------------------- 140

Seq_0  GATATTGGAAAATTCACAATGAAAGTTGTGGACGACATTCGTACCCTAAACAAGTCGGTTCATTTCCGACCATCTTGCAA 240
Seq_1  -------------------------------------------------------------------------------- 140

Seq_0  TTTTCTGAACATGAATGAGCTTTCCTCTTTGTGGGAGAAGAAGATTGGATATATGCTCCCCAGGCTCACTGTCACTGAAG 320
Seq_1  -------------------------------------------------------------------------------- 140

Seq_0  ATGACCTCCTTGCTGCAGCTGCAGAGAATATCATACCACAAGGTATCGTTGCATCATTCACACACGACATTTTCATCAAG 400
Seq_1  ------------------------AGAATATCATACCACAAAGTATCGTTGCATCATTCACACACGACATTTTCATCAAG 196

Seq_0  GGTTGTCAAGTTAATTTTTCAATTGATGGTCCAAATGAAGTTGAAGTGAGCAATCTCTATCCAGATGAAACTTTTCGAAC 480
Seq_1  GGTTGTCAAGTTAATTTTTCAATTGATGGTCCAAATGAAGTTGAAGTGAGCAATCTCTATCCAGATGAAACTTTTCGAAC 276

Seq_0  TATGGATGAATGTTTTGACGA 501
Seq_1  TATGGATGAATGTTTTGACGA 297
```

(D) Seq_2:PB.5372.3, Seq_3:PB.5372.7

```
Seq_2 AAAAACATTGGCAGAGAAAGCAGCATGGGAAGCAGCAAAAGAGAACAACATTGATTTCATTAGTATCATTCCTACATTAG 80
Seq_3 AAAAACATTGGCAGAGAAAGCAGCATGGGAAGCAGCAAAAGAGAACAACATTGATTTCATTAGTATCATTCCTACATTAG 80

Seq_2 TTGTAGGACCTTTCATAATGCCAACATTCCCACCAAGCCTAATCACTGCTCTCTCCCCCATCACTAG------------- 147
Seq_3 TTGTAGGACCTTTCATAATGCCAACATTCCCACCAAGCCTAATCACTGCTCTCTCCCCCATCACTAGTATGTATACACTT 160

Seq_2 -------------------------------------------------------------------------------- 147
Seq_3 CTCCCTCCGCAATCACCATTTTTGTGTCCATAAACTGATCATGATAATATGATTGTTTTTGTATCATAATTTTTTTGTTG 240

Seq_2 --------GAATGAAGGACACTACTCGATCATAAAGCAAGGGCAGTTTGTGCACCTTGATGATCTCTGTGAATCTCATAT 219
Seq_3 GGTGCAGGGAATGAAGGACACTACTCGATCATAAAGCAAGGGCAGTTTGTGCACCTTGATGATCTCTGTGAATCTCATAT 320

Seq_2 ATTCTTGTATGAGCGTCCTCAGGCTGAGGGCAGATACATTTGCTCCTCCCATGATGCTACCATCCATGATTTGGCCAAAC 299
Seq_3 ATTCTTGTATGAGCGTCCTCAGGCTGAGGGCAGATACATTTGCTCCTCCCATGATGCTACCATCCATGATTTGGCCAAAC 400

Seq_2 TGATGAGAGAGAAATGGCCCGAGTACAATGTCCCCACTGAGTGAGCCTCTCTATCTCTCTTCTTTCTCTACCCC 373
Seq_3 TGATGAGAGAGAAATGGCCCGAGTACAATGTCCCCACTGAGTGAGCCTCTCTATCTCTCTTCTTTCTCTACCCC 474
```

(E) Seq_0:PB.24063.3, Seq_1:PB.24063.4

```
Seq_0 CTTCCTCCTCCTGCTTCTTCTGGAGCCAAGGAAACAAACCCTGTTAGGAACTTGGAAACCAAATATATATATATATATAT 80
Seq_1 CTTCCTCCTCCTGCTTCTTCTGGAGCCAAGGAAACAAACCCTGTTAGGAACTTGGAAACCAAATATATATATATATATAT 80

Seq_0 ATATAGAGAGAGAGAGAGAGAGAGAGAGAGAGAGGGAGAGGTTGAGAGAGAAAATCTGAAGATGTTTGCAGTAGAGAATGGACT 160
Seq_1 ATATAGAGAGAGAGAGAGAGAGAGAGAGAGAGAGGGAGAGGTTGAGAGAGAAAATCTGAAGATGTTTGCAGTAGAGAATGGACT 160

Seq_0 GAAGGGAGACCCAAGGCTTGATGGCATCTCTGAAGCCATTAGAGTCATTCCTCACTTCCCTAAACCAGGAATAATGTTTC 240
Seq_1 GAAGGGAGACCCAAGGCTTGATGGCATCTCTGAAGCCATTAGAGTCATTCCTCACTTCCCTAAACCAGGAATAATGTTTC 240

Seq_0 AAGACATAACCACTTTGTTGCTGAACCACAAGGCTTTCAAGGACACTGTTGATATTTTTGTTGATAGGTACAGGTATATG 320
Seq_1 AAGACATAACCACT------------------------------------------------------------------ 254

Seq_0 GACATCTCTGTTGTTGCAGGAATAGAAGCAAGAGGATTCATGTTTGGGCCATCTATTGCCTTAGCTATTGGCGCAAAGTT 400
Seq_1 ----------------------------------------------------CTATTGCCTTAGCTATTGGCGCAAAGTT 282

Seq_0 TGTTCCATTACGTAAACCAAGAAAGTTACCAGGCAAAGT 439
Seq_1 TGTTCCATTACGTAAACCAAGAAAGTTACCAGGCAAAGT 321
```

(F) Seq_0:PB.6810.2, Seq_1:PB.6810.3

```
Seq_0 TTCCGATCTTTGCAACTTGAATCTATCTGAGTCCACCGAGAAGATCATTGCAGAGTACATATGGATCGGTGGATCTGGTA 80
Seq_1 TTCCGATCTTTGCAACTTGAATCTATCTGAGTCTACCGAGAAGATCATTGCAGAGTACATATGGATCGGTGGATCTGGTA 80

Seq_0 TGGACCTCAGAAGCAAAGCCAGGACCCTGAATGCACCAGTCTCTGATCCTTCAAAGTTACCACAATGGAACTACGATGGT 160
Seq_1 TGGACCTCAGA--------------------------------------------------------------------- 91

Seq_0 TCCAGCACTGGCCAAGCCCCTGGCGAGGACAGTGAAGTGATTTTATATCCCCAGGCAATTTATAAGGACCCATTCAGGAG 240
Seq_1 -------------------------------------------------------------------------------- 91

Seq_0 AGGCAACAACATTCTTGTAATGTGTGATGCTTACACGCCGGGTGGAGAGCCAATCCCAACAAATAAGAGGTTTGATGCTG 320
Seq_1 -GGCAACAACATTCTTGTAATGTGTGATGCTTACACGCCGGGTGGAGAGCCAATCCCAACAAATAAGAGGTTTGATGCTG 170

Seq_0 CCAAGATATTCAGCCACCCTGATGTTGTTGCTGAGGAACCTTGGTATGGTATAGAGCAGGAGTACACTTTGTTGCAGAAA 400
Seq_1 CCAAGATATTCAGCCACCCTGATGTTGTTGCTGAGGAACCTTGGTATGGTATAGAGCAGGAGTACACTTTGTTGCAGAAA 250

Seq_0 GAAGTGAAGTGGCCGATTGGTTGGCCTGTGGGAGGTTATCCTGGACCACAGGGACCATACTACTGTGGTATTGGTGCGGA 480
Seq_1 GAAGTGAAGTGGCCGATTGGTTGGCCTGTGGGAGGTTATCCTGGACCACAGGGACCATACTACTGTGGTATTGGTGCGGA 330

Seq_0 TAAAGCTTTTGGGCGAGACATTGTCGATGCCCATTATAAAGCATGTCTTTATGCTGGTATTAACATTAGTGGCATCAATG 560
Seq_1 TAAAGCTTTTGGGCGAGACATTGTCGATGCCCATTATAAAGCATGTCTTTATGCTGGTATTAACATTAGTGGCATCAATG 410

Seq_0 GAGAGGTGATGCCGGGTCAGTGGGAATTCCAAGTTGGGCCTTCTGTTGGCATCAGTTCTGGAGATCAGTTGTGGATGGC 639
Seq_1 GAGAGGTGATGCCGGGTCAGTGGGAATTCCAAGTTGGGCCTTCTGTTGGCATCAGTTCTGGAGATCAGTTGTGGATGGC 489
```

Table S1. Comparison of single molecule sequencing data of tea plant between two PacBio sequencing platforms.

Platform	Library Size	Cell Number	Reads of Insert	Read Bases of Insert (bp)	Mean Read Length of Insert (bp)	Mean Read Quality of Insert	Mean Number of Passes
PacBio Sequel (This study)	1-6K	3	1,388,066	2,446,983,161	1,762	0.92	10
PacBio RS II (Xu et al. 2017)	<1K	2	55,037	42,298,256	768	0.96	33
	1-2K	2	135,732	293,235,156	2,160	0.94	11
	2-3K	2	119,629	361,701,882	3,023	0.91	9
	3-6K	1	51,549	200,280,602	3,885	0.88	4
